# Establishment of local diagnostic reference levels for trunk computed tomography examinations at governmental hospitals in the Gaza Strip: a cross-sectional study

**DOI:** 10.1007/s12194-026-01053-x

**Published:** 2026-04-30

**Authors:** Amjad Ayyad, Yasser Alajerami, Ahmed Najim, Husam H. Mansour, Muhammad Khalis Abdul Karim, Fahad Alghamdi, Kinan Mokbel

**Affiliations:** 1 Department of Medical Imaging, Faculty of Applied Medical Sciences, Al- Azhar University, Gaza Strip, Palestine; 2 Department of Nursing, Faculty of Applied Medical Sciences, Al-Azhar University, Gaza Strip, Palestine; 3 Department of Health and Care Professions, Faculty of Health and Life Sciences, University of Exeter, Exeter, UK; 4 Department of Physics, Faculty of Science, Universiti Putra Malaysia, Serdang, Selangor Malaysia; 5 Department of Radiologic Technology, College of Applied Medical Sciences, Qassim University, Buraydah, Saudi Arabia

**Keywords:** Diagnostic reference levels, Local diagnostic reference level, Computed tomography, Trunk CT, Radiation dose optimisation, Volumetric CT dose index, CTDI_vol_ Dose-length product, DLP, Gaza Strip

## Abstract

To establish the local diagnostic reference levels (LDRLs) for trunk multi-slice CT (MSCT) examinations in the Gaza Strip, Palestine. This cross-sectional study included adult oncology patients undergoing trunk MSCT at two governmental hospitals in Gaza Strip; Al Shifa Medical Complex (SMC) and Al Aqsa Martyrs Hospital (AMH), using an adapted dose survey booklet. Data collected from July 2019 to March 2020 included patient characteristics, volumetric CT dose index (CTDI_vol_) and dose length product (DLP). Descriptive, univariate and multivariate analyses identified key factors affecting radiation dose, and the coefficient of variation between scanner- and software-derived dose values was also determined. A total of 170 trunk CT examinations were analysed (57.1% SMC, 42.9% AMH). 72.94% were female; the mean age of the participants is 53.1 ± 15.8 years, and the mean body mass index was 30 ± 6.1. The estimated LDRLs for trunk CT were 13 mGy for CTDI_vol_ and 1010.4 mGy·cm for DLP. There was notable variation between hospitals in CTDI_vol_ and DLP (*p* < 0.001). At SMC, factors such as tube current, peak kilovoltage, scan length, pitch and BMI significantly affected dose indices. In contrast, at AMH, the main influences were tube current and scan length. CTDI_vol_ had a greater impact on DLP than scan length at both locations. LDRLs for trunk CT scans in the Gaza Strip were established and found to be generally comparable to international benchmarks. Notable variation in doses between hospitals indicates potential for improvement through standardising protocols, managing scan lengths and using techniques tailored to patient size.

## Introduction

Computed tomography (CT) plays a crucial role in modern diagnostics and follow-up care. The rapid advances in Multi-Slice Computed Tomography (MSCT) scanners make it a valuable diagnostic tool, leading to a surge in CT exam requests [1]. However, it accounts for a significant portion of medical radiation exposure relative to other imaging methods, highlighting the importance of optimisation as a key aspect of radiological governance [2–4]. Trunk CT examinations, including chest–abdomen–pelvis imaging, are common in clinical practice and are primarily used for cancer staging and follow-up. Because trunk CT examinations involve large scan ranges and are often performed repeatedly, they significantly contribute to the collective effective dose (ED) from medical imaging and constitute a major part of the population’s radiation burden from diagnostic CT [5]. Technological improvements in CT have reduced radiation dose, but significant hospital-level differences remain [6–8]. To address this and improve dose optimisation, the International Commission on Radiation Protection (ICRP) introduced Diagnostic Reference Levels (DRLs) [9].

DRLs are widely recognised as a practical optimisation tool for identifying unusually high patient dose levels for a given examination and for prompting protocol review and standardisation [9]. In a dose survey, the third quartile value of each examination, where 75% of the data is below it, is used as the DRL acceptance criterion [10, 11]. Regional and international DRLs are inadequate because of differences in region-specific training, equipment and populations used to establish them [12]. The applicability of international DRLs to local settings is limited by differences in patient habits, CT scanner generations, and protocol philosophies. Patient body composition, such as higher BMI, affects dose control. Older scanners lack advanced dose-reduction tech, leading to higher doses. Protocols vary across regions, affecting scan length and image quality. These factors highlight the need for local reference levels grounded in real-world practice. Many countries have established local and national DRLs for radiological examinations, with reviews showing substantial dose reductions (16%-30%) from their use [13–15].

In the Gaza Strip, access to advanced imaging is constrained by limited infrastructure, with CT services concentrated in a small number of governmental hospitals. At the time of data collection, fewer than ten operational CT scanners served the entire Gaza Strip, with high-volume oncology-related examinations predominantly referred to major centres such as Al Shifa Medical Complex (SMC) and Al Aqsa Martyrs Hospital (AMH). This centralisation, combined with restricted equipment availability, maintenance challenges, and the absence of unified imaging protocols, places additional pressure on existing CT services and complicates dose optimisation efforts. Patient dose can be minimised through proper protocol selection, scanning parameters and patient positioning. Developing countries like Palestine face limited equipment, poor maintenance and weak radiation protection. Radiological centres in Gaza may face challenges related to the consistency of equipment calibration, optimisation of radiation exposure and standardisation of imaging protocols.

Palestine currently lacks national diagnostic reference levels for CT scans, relying instead on individual protocols without external benchmarks. This highlights the need for this study, which offers the first evidence-based local diagnostic reference levels for trunk CTs in Gaza. Establishing a local diagnostic reference level (LDRL) is essential for dose optimisation. DRL can be established at local, regional, and national levels. A local LDRL uses data from a few institutions, reflecting site-specific equipment, protocols, and patient groups. In contrast, a national diagnostic reference level (NDRL) requires broad data from many healthcare facilities and scanner types nationwide. Currently, in the absence of a coordinated dose registry, limited CT scanners, and diverse equipment, establishing a national DRL is not feasible. Therefore, the aim of this study was to establish LDRLs for trunk multi-slice CT examinations at two governmental hospitals in the Gaza Strip. A secondary aim was to identify scan-related factors associated with variation in radiation dose. The LDRL can guide MSCT procedures to manage and optimise dose, ensuring it is appropriate for clinical needs [16]. Many organisations support the use of DRLs as a dose-optimisation tool [17].

## Materials and methods

### Study design and ethics approval

**Fig. 1:**
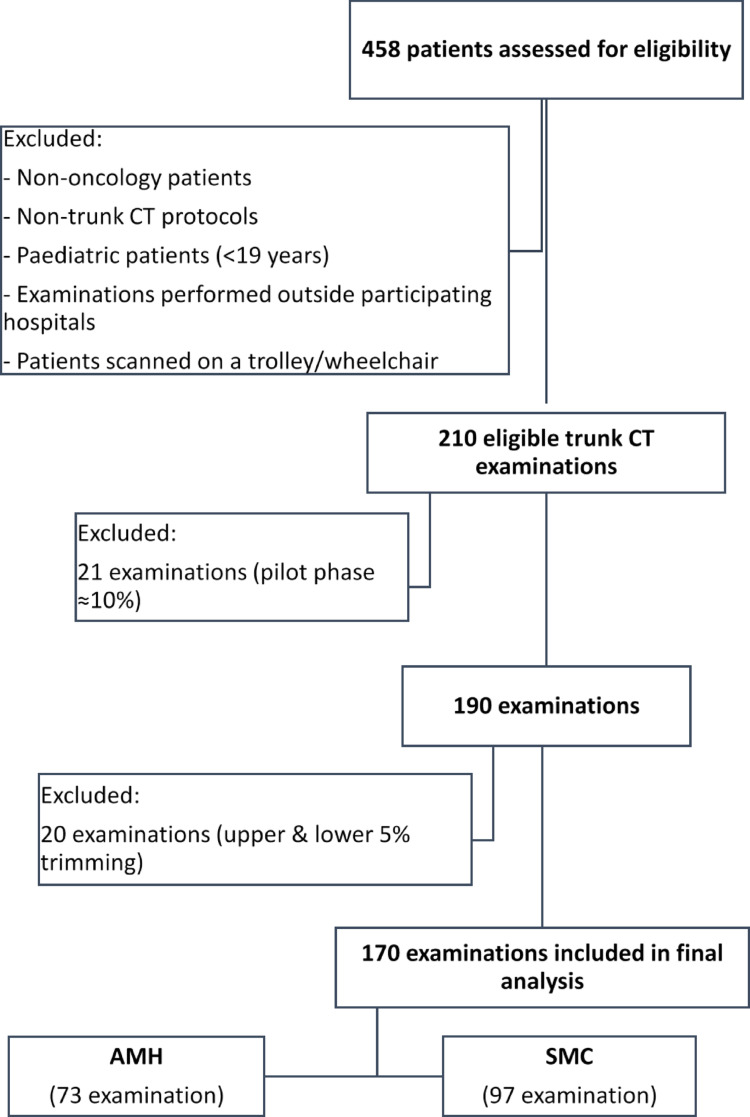
Flowchart of patient and examination inclusion and exclusion

In this cross-sectional study, data were obtained from 458 patients at two hospitals in the Gaza Strip: Al Shifa Medical Complex (SMC) and Al Aqsa Martyrs Hospital (AMH). A total of 458 patients were assessed for eligibility during the study period. Patients were screened according to predefined inclusion criteria, including adult age (≥ 19 years), oncology-related clinical indication, and completion of a trunk MSCT examination at one of the participating hospitals. Patients were excluded if they were non-oncology cases, underwent MSCT protocols other than trunk examinations, were scanned outside the participating centres, or attended in a wheelchair or on a trolley for ethical reasons. Application of these criteria yielded 210 eligible trunk CT examinations. Approximately 10% of these were excluded during a pilot phase conducted to refine data-collection procedures, resulting in 190 examinations. To minimise the influence of extreme values, pairwise trimming of the upper and lower 5% of dose metrics was then performed in accordance with ICRP recommendations, yielding a final analytical dataset of 170 examinations, as illustrated in Fig. 1. Ethical approval was granted by the Helsinki Committee (HC) for Ethical Approval, Palestinian Health Research Council (PHRC), Gaza Strip, Palestine (approval number PHRC/HC/572/19; 17 June 2019). Administrative approval was obtained from the Ministry of Health, State of Palestine (Human Resources Development Directorate; correspondence number 381554; 17 Oct 2019). Informed consent was obtained from all participants before participation. The CT machine specifications used in this study are as follows: At SMC, imaging was performed on a Philips HealthCare Ingenuity Core 128™ scanner with 128 slices, manufactured in October 2013 and installed in May 2015. At AMH, a General Electric Health Care Optima CT540 scanner with 16 slices was used; it was manufactured in February 2016 and installed in March 2017. Data was collected between July 2019 and March 2020.

### Data collection tools

Data was collected for each trunk CT examination performed for the specified clinical indication, oncology follow-up, to standardise data capture and reduce protocol-related heterogeneity, as different clinical indications may require different acquisition protocols, even when imaging the same anatomical region. Dose and scan-parameter data were recorded at two points: first, during protocol selection, and second, immediately after the examination. This enabled cross-checking against the scanner’s dose report at the end of each scan.

Patient demographic information, acquisition parameters and dose indices were collected using an adapted, self-administered Canadian CT dose survey booklet (Supplement, S1). The following parameters were extracted from the CT console: tube potential (kV), tube current (mA), rotation time (s), tube current–time product (mAs), beam collimation (N×hcol), table feed, slice thickness, scan length, pitch, CTDI_vol_ and dose length product (DLP). The ED values were calculated across the surveyed hospitals primarily because of differences in the DLP values used for the examinations studied, and for general comparison with other imaging procedures, but were not used for establishing the DRL or as the primary outcome in dose optimisation analyses, as the study focused on establishing LDRLs for dose optimisation [18].

The factors affecting CTDI_vol_ and DLP values at the two hospitals are illustrated in Table 1. The mean peak kilovoltage in SMC is 120kVp (range: 100- 140kVp), whereas in AMH it is fixed at 120kVp. Regarding tube current, the mean in SMC is 285.3 mA (range: 266-400 mA), whereas in AMH it is 515.9 mA (range: 500-733.3 mA). Additionally, the mean tube current-time product is 214 mAs (range: 200-300mAs), whereas in AMH it is 309.59mAs (range: 300-400mAs). The mean pitch is 0.99 (range: 0.83-1.14 ) in SMC, whereas in AMH it is fixed at 1.37. For scan length, the mean is 667 mm (range: 570–850 mm) in SMC and 708 mm (range: 565–951 mm) in AMH.

**Table 1:** Parameters Affecting the CTDI_vol_ and DLP Values (*n* = 170)

Variable	SMC	AMH
	Mean ± SD	Mean ± SD
Peak kilovoltage (kVp)	120.41 ± 4.98	120 (fixed)
Tube current (mA)	285.35 ± 30.95	515.98 ± 59.34
Current time product (mAs)	214.02 ± 23.21	309.59 ± 35.61
Pitch	0.99 ± 0.04	1.37 (fixed)
Scan length (mm)	667 ± 56.9	708 ± 70.9
Rotation time (s)	0.75 (fixed)	0.60 (fixed)
Number of rows	64 (fixed)	16 (fixed)
Nominal slice thickness (mm)	0.625 (fixed)	1.250 (fixed)
Beam Collimation (mm)‡	40 (fixed)	20 (fixed)
Reconstructed slice thickness (mm)	2 (fixed)	2.5 (fixed)
Gantry Tilt	0 (fixed)	0 (fixed)
Effective dose (mSv)	15.6	12

*SMC* Al Shifa Medical Complex, *AMH* Al Aqsa Martyrs Hospital, *SD* standard deviation

‡ (detector Configuration product)

CT-Expo dose calculation software (version 2.3.1; Germany) was subsequently used to validate and compare the dose values reported by the CT scanners, thereby assessing the reliability of console-displayed dose metrics. The validation of scanner-reported dose values was performed retrospectively. First, all relevant scanning parameters were extracted from the console or DICOM metadata. These parameters were then used as inputs in the CT-Expo software. The accuracy of the scanner-reported dose was verified by comparing the console values against the software-generated results. Furthermore, CT-Expo was utilised to compute specific organ doses based on these validated parameters. When scanner- and protocol-specific inputs are provided, CT-Expo outputs CTDI_vol_ and the corresponding DLP and estimates ED (and organ doses) using ICRP publications 60 or 103 tissue-weighting factors [19, 20].

### Statistical analysis

Microsoft Excel (Microsoft, Redmond, WA, USA) and SPSS Statistics version 23.0 (SPSS Inc., Chicago, IL, USA) were used for data management and statistical analysis. The initial sample comprised 210 examinations. Following the exclusion of 20 examinations during a preliminary pilot phase, the analytical dataset comprised 190 examinations. A 10% trimming approach was subsequently applied to minimise the influence of extreme dose values. This method reduces distortion of the dose distribution caused by outliers, which may arise from atypical patient anatomy or non-standard scanning protocols, thereby providing a more robust and representative estimate of typical clinical practice. Specifically, the upper and lower 5% of outcome values were excluded using pairwise trimming, reducing the final analytical dataset to 170 examinations. This approach is consistent with established methodologies used in DRL development and dose optimisation studies and aligns with ICRP guidance [16].

Descriptive statistics (frequency tables, scatterplots and figures) were used to summarise the data. ED was estimated from the DLP using ED (mSv) = K×DLP, where K = 0.015 for trunk (chest-abdomen-pelvis) CT [21]. DRLs for CTDI_vol_ and DLP were defined as the 75th percentile (third quartile) of their distributions. Between hospital differences in outcome measures were assessed using an independent samples t-test. Pearson’s correlation coefficient was used to examine associations between scan parameters and outcome variables (CTDI_vol_ and DLP). Multiple linear regression was performed to identify predictors of CTDI_vol_ and DLP. Statistical significance was set at *p* < 0.05 with 95% confidence intervals. In addition, the coefficient of variation (CV) between scanner-reported and software-calculated dose values was computed to quantify relative agreement. A CV within about ± 20% was acceptable for CT dose index display accuracy, while higher values suggested potential clinical issues relevant [22].

## Results

### Patient characteristics and trunk CT scan parameters

During the second half of the data collection period, the CT scanner at Al-Rantesi Hospital and European Gaza Hospital (EGH) malfunctioned. Consequently, patients who would normally have undergone CT examinations at EGH were referred to AMH for scanning on the AMH CT scanner, using standard AMH acquisition protocols. All examinations performed during this period were therefore analysed as AMH cases based on the scanner and protocol used. No CT examinations acquired on the EGH scanner during the malfunction period were included in the final dataset. Overall, trunk CT examinations were distributed across the surveyed hospitals as follows: 97 cases (57.1%) were performed at SMC and 73 cases (42.9%) at AMH. Participants’ characteristics, as shown in Table 2, indicate that 27% are male, and 73% are female. The average age of the patients examined is 53.1 ± 15.8 years, and the overall mean body mass index (BMI) was 30.0 ± 6.1 kg/m². The BMI mean for SMC was 30.4 ± 5.5 kg/m², whereas in AMH it was 29.5 ± 6.9 kg/m².

**Table 2:** Distribution of Participants Characteristics (*n* = 170)

Variable	Total	SMC	AMH
Total, n (%)	170 (100)	97 (57.1)	73(42.9)
Female, n (%)	124 (72.9)	72 (74.2)	52 (71.2)
Male, n (%)	46 (27.1)	25 (25.8)	21 (28.8)
Age (Mean ± SD)	53.1 ± 15.8	53.3 ± 15.5	52.8 ± 16.2
BMI (Mean ± SD)	30.0 ± 6.1	30.4 ± 5.5	29.5 ± 6.9

*SMC* Al Shifa Medical Complex, *AMH* Al Aqsa Martyrs Hospital, *BMI* body mass index, *SD* standard deviation

### Inter-hospital dose variation and local diagnostic reference levels

The calculated DRLs obtained from the two hospitals, based on CTDI_vol_ and estimated LDRL for trunk CT examinations, were expressed as the third quartile (75th percentile) of the CTDI_vol_ and DLP values, as shown in Table 3. There were statistically significant differences in CTDI_vol_ and DLP between the two hospitals surveyed. The mean CTDI_vol_ was higher in SMC than in AMH (14.2 vs. 11.7 mGy, *p* < 0.001). Additionally, the mean DLP was higher in SMC than in AMH (1041.6 vs. 799.3 mGy.cm, *p* < 0.001).

**Table 3:** Differences in CTDI_vol_ and DLP values between the surveyed hospitals and pooled local diagnostic reference levels (75th percentile) for trunk CT examinations.

CT Parameters	Hospital	*N*	Mean ± SD	*p*	Pooled 75th percentile (local DRL)
CTDI_vol_ (mGy)	SMC	97	14.2 ± 2.5	< 0.001*	13.0
	AMH	73	11.7 ± 0.5		
DLP (mGy.cm)	SMC	97	1041.6 ± 201.8	< 0.001*	1010.4
	AMH	73	799.3 ± 68.2		

*kVp* kilovolt peak,* mGy* milligrays, * mGy.cm* milligrays per centimetre, *SMC* Al Shifa Medical Complex, *AMH* Al Aqsa Martyrs Hospital, *SD* standard deviation

* Statistically significant *p* < 0.05 Note: The 75th percentile values represent the pooled local diagnostic reference level calculated from the combined dataset across both hospitals, not hospital-specific quartiles.

The 75th percentile values obtained represent CT practice in the Gaza Strip and can be used to compare with related regional surveys, recommended standards and other countries. The calculated DRLs for the two hospitals are based on CTDI_vol_ of 13 mGy and DLP of 1010.4 mGy.cm. The coefficient of variation for CTDI_vol_ and DLP was lower in SMC (approximately 11% and 2%, respectively) compared with AMH, where higher variability was observed (approximately 22% for CTDI_vol_ and 29% for DLP). Furthermore, the mean difference between CT-Expo and console-reported CTDI_vol_ was 0.38 mGy (95% CI: 0.19–0.58) at SMC and 4.40 mGy (95% CI: 4.05–4.74) at AMH. For DLP, the mean differences were 30.70 mGy·cm (95% CI: 14.36–47.03) at SMC and 414.89 mGy·cm (95% CI: 370.59–459.19) at AMH, indicating reduced agreement between console-reported and software-calculated dose values at AMH. Across the full dataset, Pearson correlation analysis showed statistically significant positive associations between scanner-reported and software-calculated values for CTDI_vol_ (*r* = 0.574, *p* < 0.001), DLP (*r* = 0.419, *p* < 0.001), and ED (*r* = 0.407, *p* < 0.001).

### Influence of scan parameters on dose indices

Table 4 presents Pearson correlation analyses between scan parameters and radiation dose indicators across the studied hospitals. At SMC, peak kilovoltage showed a strong positive correlation with CTDI_vol_ (*r* = 0.772, *p* < 0.001) and a moderate positive correlation with DLP (*r* = 0.623, *p* < 0.001), indicating that increases in tube voltage were associated with higher radiation dose metrics. Tube current was also strongly correlated with both CTDI_vol_ (*r* = 0.701, *p* < 0.001) and DLP (*r* = 0.706, *p* < 0.001), confirming its substantial influence on patient radiation dose. Pitch showed a statistically significant inverse association with CTDI_vol_ (*r* = − 0.358, *p* < 0.001) and DLP (*r* = − 0.273, *p* = 0.007), suggesting that higher pitch values were associated with reduced dose indices. BMI showed moderate positive correlations with CTDI_vol_ and DLP at SMC (*r* = 0.469 and 0.375, respectively; *p* < 0.001). At AMH, tube current exhibited a strong correlation with CTDI_vol_ (*r* = 0.798, *p* < 0.001) but weakly with DLP (*r* = 0.455, *p* < 0.001). At the same time, BMI showed no significant association with either dose metric. Partial correlation analysis showed that DLP was strongly associated with CTDI_vol_ and scan length at SMC (*r* = 0.978 and 0.911; *p* < 0.001) and moderately associated at AMH (*r* = 0.650 and 0.636; *p* < 0.001). In both hospitals, the association between DLP and CTDI_vol_ was stronger than that with scan length, indicating CTDI_vol_ as the dominant contributor to DLP.

**Table 4:** Correlation between the Outcome Variables and the Independent Variables

Variables	SMC				AMH			
	CTDI_vol_		DLP		CTDI_vol_		DLP	
	*r*	*p*	*r*	*p*	*r*	*p*	*r*	*p*
Peak Kilovoltage	0.772^*^	< 0.001	0.623^*^	< 0.001	*–*	*–*	*–*	*–*
Tube Current	0.701^*^	< 0.001	0.706^*^	< 0.001	0.798^*^	< 0.001	0.455^*^	< 0.001
Pitch	− 0.358^*^	< 0.001	− 0.273^*^	0.007	*–*	*–*	*–*	*–*
BMI	0.469^*^	< 0.001	0.375^*^	< 0.001	-0.033	0.783	− 0.031	0.793
Scan length‡	*–*	*–*	0.911^*^	< 0.001	*–*	*–*	0.636^*^	< 0.001
CTDI_vol‡_	*–*	*–*	0.978^*^	< 0.001	*–*	*–*	0.650^*^	< 0.001

*SMC* Al Shifa Medical Complex,* AMH* Al Aqsa Martyrs Hospital, *BMI* body mass index

‡: Partial Correlation between DLP and Each of CTDI_vol_ and Scan Length * Statistically significant *p* < 0.05

### Beam collimation, tube current modulation and protocol standardisation

The effects of beam collimation on CTDI_vol_ and DLP across the two hospitals are summarised in Table 5. Beam collimation was associated with statistically significant differences in both dose metrics. CTDI_vol_ was higher at SMC with 40 mm collimation (14.2 ± 2.5mGy) than at AMH with 20 mm collimation (11.7 ± 0.5 mGy; *p* < 0.001). Similarly, DLP was significantly greater at SMC (1041.6 ± 201.9 mGy.cm) than at AMH (799.3 ± 68.2 mGy.cm; *p* < 0.001). For automated tube current modulation (TCM); there were no statistically significant differences in CTDI_vol_ or DLP between TCM used versus not used within either hospital (SMC: CTDI_vol_ *p* = 0.640, DLP *p* = 0.584; AMH: CTDI_vol_ *p* = 0.058, DLP *p* = 0.154) (Table 6).

**Table 5:** The Effect of Beam Collimation on the Resultant CTDI_vol_ and DLP

Variables			CTDI_vol_		DLP	
		*n*	Mean ± SD	*p*	Mean ± SD	*p*
Beam collimation						
SMC	40 mm	97	14.2 ± 2.5	< 0.001*	1041.6 ± 201.9	< 0.001*
AMH	20 mm	73	11.7 ± 0.5		799.3 ± 68.2	

*SMC* Al Shifa Medical Complex, * AMH* Al Aqsa Martyrs Hospital, * CTDI*_vol_ volumetric CT dose index, * DLP*: dose length product, * n* number of participants from each hospital, *SD* standard deviation

**Table 6:** The Effect of Tube Current Modulation on the Resultant CTDI_vol_ and DLP

Variables			CTDI_vol_		DLP	
		*n*	Mean ± SD	*p*	Mean ± SD	*p*
Tube Current Modulation						
SMC	Used	1	13.04	0.640	930.8	0.584
	Not used	96	14.2 ± 2.5		1042.8 ± 202.6	
AMH	Used	6	12.89 ± 1.3	0.058	882.02 ± 130.9	0.154
	Not used	67	11.59 ± 0.000		791.87 ± 55.7	

*SMC* Al Shifa Medical Complex, * AMH* Al Aqsa Martyrs Hospital, *CTDI*_vol_ volumetric CT dose index, * DLP* dose length product, *n* number of participants from each hospital, *SD* standard deviation

Multivariable linear regression was performed to identify scan parameters independently associated with CTDI_vol_ and DLP at SMC and AMH (Table 7). At SMC, tube current (B = 0.047, 95% CI: (0.042;0.052); *p* < 0.001) and peak kilovoltage (B = 0.340, 95% CI: (0.311;0.370); *p* < 0.001) were significant independent predictors of CTDI_vol_. At AMH, only tube current remained significantly associated with CTDI_vol_ (B = 0.007, 95% CI: (0.006;0.008); *p* < 0.001). At SMC, tube current (B = 3.452, 95% CI: (2.999;3.905); *p* < 0.001), peak kilovoltage (B = 23.431, 95% CI: (20.722;26.140); *p* < 0.001), and scan length (B = 1.435, 95% CI: (1.207;1.664); *p* < 0.001) were independently associated with increased DLP. At AMH, DLP was significantly influenced by tube current (B = 0.475, 95% CI: (0.225;0.724); *p* < 0.001) and scan length (B = 0.213, 95% CI: (0.002;0.423); *p* = 0.048).

**Table 7:** Multivariate Analysis for Factors Affecting CTDI_vol_ and DLP

Variable	SMC		AMH	
	B (95% CI)	*p*	B (95% CI)	*p*
CTDI_vol_				
Constant	− 37.44 (− 42.9;− 31.89)	< 0.05	8.146 (7.158;9.133)	< 0.05
Body mass index	− 0.005 (− 0.035;0.025)	0.735	0.006 (− 0.005;0.017)	0.259
Tube current	0.047 (0.042;0.052)	< 0.001*	0.007 (0.006;0.008)	< 0.001*
Scan length	0.000 (− 0.003;0.002)	0.722	0.000 (− 0.001;0.001)	0.660
Pitch	− 2.410 (− 6.040;1.219)	0.190	–	
Peak kilovoltage	0.340 (0.311;0.370)	< 0.001*	–	
DLP				
Constant	− 3584.565 (− 4096.53;-3072.61)	< 0.05*	386.284 (189.808;582.759)	< 0.05*
Body mass index	0.289 (− 2.472;3.049)	0.836	0.587 (− 1.534;2.709)	0.582
Tube current	3.452 (2.999;3.905)	< 0.001*	0.475 (0.225;0.724)	< 0.001*
Scan length	1.435 (1.207;1.664)	< 0.001*	0.213 (0.002;0.423)	0.048*
Pitch	− 148.433 (− 483.347;186.481)	0.381	–	
Peak kilovoltage	23.431 (20.722;26.140)	< 0.001*	–	

*SMC* Al Shifa Medical Complex, *AMH* Al Aqsa Martyrs Hospital, *CTDI*_vol_ volumetric CT dose index, * DLP* dose length product, *B* regression coefficient, *CI* confidence interval

* Statistically significant *p* < 0.05, CI = 95%

## Discussion

This study established LDRLs for trunk CT exams at two hospitals in the Gaza Strip, with key factors influencing radiation dose. The findings also offer a snapshot of current CT practice and a basis for dose optimisation and future DRL development. Trunk CT exams made up a significant part of CT activity in the surveyed hospitals, mainly for oncology follow-up and lymphoma staging. SMC conducted more exams than AMH, reflecting Gaza City’s concentrated oncology services and cancer trends across the Gaza Strip [23]. The mean BMI shows a mostly overweight to obese group; this high BMI distribution affects dose optimisation, as patient size influences exposure and radiation dose [24]. Although the LDRLs in this study were from a patient group with a mean BMI of around 30, the CTDIvol and DLP values were similar to or lower than those in several international studies. The estimated ED was 15.6 mSv at SMC and 12 mSv at AMH, reflecting the higher CTDI_vol_ and DLP values observed at SMC, which were mainly due to variations in scan parameters such as beam collimation and tube current. This is a general indication of radiation risk and should be interpreted with caution, as it represents a population-based estimate rather than a patient-specific dose measurement [25].

This discrepancy may stem from fixed or semi-fixed scanning settings, including tube voltage and reference tube current, which may limit dose escalation in larger patients when automatic exposure control is conservatively configured or not fully optimised for patient size. Scanner type and manufacturer-specific dose modulation can also influence dose efficiency. Additionally, tighter control of scan length and coverage can substantially reduce DLP, even in patients with higher BMI, thereby partially offsetting the dose increases associated with larger body habits. Conversely, reliance on standardised protocols not fully tailored to patient physique may result in similar dose indices across diverse patients. These findings illustrate the complex relationship between patient factors, protocol design, and scanner technology in CT radiation dosing.

The parameters that remained fixed within each hospital were not evaluated as sources of within-hospital variation in CTDI_vol_ or DLP. The rotation time, number of rows, nominal slice thickness, beam collimation, reconstructed slice thickness and no tilting were recorded in all exams. Finally, the mean ED values are SMC 15.6 mSv and AMH 12mSv. The calculated LDRLs for trunk CT exams, CTDI_vol_ (13 mGy); DLP (1010.4 mGy.cm), reflect current practice across the two hospitals and fall within the range reported internationally. These values were compared with DRLs from regional and international DRL reports, including the UK, Canada, the USA, Libya, Saudi Arabia, Singapore, Australia, and Japan [26, 27]. The CTDI_vol_ LDRL is comparable to the UK value (13 mGy) and lower than that reported in the USA (15 mGy), indicating broadly similar dose optimisation practices for tube current modulation and image quality targets. In contrast, the DLP LDRL (1010.4 mGy·cm) is slightly higher than values reported in the UK (1003 mGy·cm) and the USA (947 mGy·cm), but remains lower than those reported in several other countries. Notably, both CTDI_vol_ and DLP in this study are higher than those reported in Singapore (13 mGy and 1010.4 mGy·cm vs. 12 mGy and 823 mGy·cm). These intercountry differences likely reflect variations in patient body habitus, protocol philosophy, scanner generation, and scan length selection. In particular, longer scan coverage and less stringent control of anatomical scan extent may contribute to higher DLP values despite comparable CTDIvol, as DLP is directly influenced by scan length as well as tube output. The agreement at SMC shows console-reported CTDI_vol_ and DLP are reliable for dose monitoring. Greater variability in AMH suggests that relying solely on these metrics may misestimate patient dose, thereby affecting protocol optimisation and benchmarking. Regular cross-validation is important, especially with older scanners or less standardised protocols.

On the other hand, significant differences in CTDI_vol_ and DLP appeared between SMC and AMH, with SMC showing higher dose indices despite AMH using higher tube current. Variations likely stem from differences in scanner type, operator protocols and scanning parameters. This underscores the multifactorial nature of CT radiation dose, influenced by scanner characteristics, beam collimation, pitch, scan length and protocols. These findings align with prior studies highlighting hospital variability for the same exams, yet emphasising the need for local DRLs over international standards [28, 29].

Our correlation and multivariable analyses confirm that tube current and voltage mainly affect CTDI_vol_, especially at SMC, where protocols vary in kVp. The strong positive associations observed are consistent with prior CT research, demonstrating that automated kVp selection reduces CTDI_vol_ compared with fixed kVp protocols while maintaining diagnostic image quality in oncology CT examinations [30, 31]. In addition, tube current is directly linearly related to radiation dose [32]. At AMH, with fixed kVp and pitch, tube current remains the main predictor, indicating limited protocol customisation.

Our findings demonstrate that scan length was a key predictor of DLP in both hospitals, highlighting its role in total dose. While CTDI_vol_ had a stronger link, excessive or unclear scan ranges can raise patient exposure without additional benefit. Longer scan lengths in surveyed hospitals likely explain the higher DLP compared to international DRLs, such as Singapore [33]. Furthermore, pitch showed an inverse relationship with dose at SMC; however, this effect disappeared in multivariable modelling. This aligns with modern multislice CT systems, where automatic tube current adjustment compensates for pitch changes, resulting in minimal dose reduction [34]. BMI was significantly associated with dose at SMC but not at AMH, suggesting patient size was not well integrated into protocols at AMH. This underscores the need for BMI adapted protocols to ensure consistent image quality and prevent unnecessary dose increases.

In the present study, beam collimation showed significant differences in CTDI_vol_ and DLP between hospitals, but its impact on dose variation was minor. While beam collimation was fixed within each hospital, it differed between hospitals. The higher dose indices at SMC are more likely due to differences in tube voltage, scan length, and scanner design rather than collimation alone. This aligns with prior studies indicating that in modern multi-detector CTs, radiation dose depends more on kVp, tube current, pitch, and scan length than on collimation [34, 35]. TCM did not show a significant dose reduction in either hospital, unlike the established literature [36]. However, TCM activation wasn’t systematically recorded during exams. Hence, the lack of observed effect may result from inconsistent use, setup, patient centering, or operator experience, not an inherent lack of dose-saving potential [37–40].

This study has, however, some limitations. It was conducted at only two hospitals and included only adult oncology patients, which could limit the applicability of the findings to other clinical settings and populations. CT services in the Gaza Strip are delivered through a limited number of governmental hospitals, with high-volume and oncology-related CT examinations commonly referred to central facilities. The two hospitals included in this study are among the principal public providers of CT imaging and receive referrals from across the Gaza Strip. Although inclusion of additional centres would enhance generalisability, the practices evaluated are likely representative of routine governmental CT practice in this setting. Furthermore, variations in CT scanner models and fixed protocol parameters restricted the evaluation of certain dose determinants. In addition, image quality was not systematically assessed; therefore, dose optimisation was evaluated based on radiation dose metrics alone without direct consideration of diagnostic image quality. Also, the cross-sectional design prevents assessment of long-term dose optimisation and removing extreme values may have excluded some high-dose examinations.

## Conclusions

This study established LDRLs for trunk CT examinations at two governmental hospitals in the Gaza Strip, providing the first evidence-based benchmarks for dose optimisation in this setting. Significant inter-hospital variation in CTDI_vol_ and DLP was observed, driven primarily by modifiable scan parameters rather than patient characteristics. Tube current, tube voltage and scan length were identified as the main determinants of radiation dose, with CTDI_vol_ contributing more strongly to DLP than scan length. Although the derived LDRLs are broadly comparable to international values, the findings indicate opportunities for further optimisation, particularly through improved protocol standardisation and scan-range control. The results support the use of LDRLs as a practical tool for dose monitoring, optimisation and the future development of national diagnostic reference levels in Palestine.

## Data Availability

Data supporting the findings of this study are included in the article and Supplementary Materials; further data are available from the corresponding author upon reasonable request.

## Rights Retention Statement

For the purpose of open access, the authors have applied a Creative Commons Attribution (CC BY) licence to any Author Accepted Manuscript version arising from this submission.
